# Effects of galvanic vestibular stimulation on motor control in patients with Parkinson's disease: study protocol

**DOI:** 10.1055/s-0046-1816041

**Published:** 2026-02-27

**Authors:** Catarina Costa Boffino, Tali Yael Holzhacker, Sonia Maria Azevedo Silva, Michael C. Schubert, Flávia Doná

**Affiliations:** 1 Instituto de Assistência Médica ao Servidor Público Estadual de São Paulo, Hospital do Servidor Público Estadual (IAMSPE-HSPE), São Paulo SP, Brazil.; 2 Universidade de São Paulo, Faculdade de Medicina, Hospital das Clínicas, Instituto de Psiquiatria, Laboratório de Psicopatologia e Terapêutica Psiquiátrica, São Paulo SP, Brazil.; 3 Universidade Municipal de São Caetano do Sul, Departamento de Saúde, São Caetano do Sul SP, Brazil.; 4 Universidade Cruzeiro do Sul, Faculdade de Fisioterapia, São Paulo SP, Brazil.; 5 Instituto de Assistência Médica ao Servidor Público Estadual de São Paulo, Hospital do Servidor Público Estadual (IAMSPE-HSPE), Setor de Distúrbio do Movimento, São Paulo SP, Brazil.; 6 Johns Hopkins University, School of Medicine, Department of Otolaryngology Head and Neck Surgery, Laboratory of Vestibular Neuroadaptation, Baltimore MD, United States of America.; 7 Johns Hopkins University, School of Medicine, Department of Physical Medicine and Rehabilitation, Baltimore MD, United States of America.

**Keywords:** Parkinson Disease, Transcranial Direct Current Stimulation, Gait, Postural Balance

## Abstract

**Background:**

Parkinson's disease (PD) presents with both motor and non-motor symptoms. Postural and gait aspects, as well as the risk of falls, are important causes of morbidity and mortality in PD. The neural correlates of PD are alterations in the substantia nigra of the midbrain and, among other sites mentioned, the pontine peduncle nucleus stands out. Noninvasive neuromodulation, such as galvanic vestibular stimulation (GVS), can be used in the neural centers involved in motor control alterations in PD. Projections from the vestibular system are involved in motor control and can stimulate the basal nuclei and the pontine peduncle nucleus, as well as strengthen neural networks.

**Objective:**

To analyze the effects of GVS associated with physical-functional exercise on the motor control of PD patients.

**Methods:**

A randomized, placebo-controlled clinical trial. Participants with PD, diagnosed through the Hoehn & Yahr scale (HY) 2 or 3, will be allocated to groups. Pre- and postintervention and follow-up assessments will follow a structured protocol of physical-functional instruments, as recommended in the European Guidelines for managing people with PD. The intervention for both groups will follow the American Neurofunctional Physical Therapy Guideline for managing people with PD.

**Results:**

It is expected that, in the experimental group, the exercises will be associated with active GVS. Finally, a descriptive and statistical analysis must be conducted to verify the effects of GVS.

**Conclusion:**

The study of new devices focusing on motor control in PD is a novel approach and warrants further investigation in the context of vestibular function.

## INTRODUCTION

Parkinson's disease (PD) is a chronic and progressive neurodegenerative disorder characterized clinically by four cardinal signs (tremor, bradykinesia, rigidity, and postural instability) resulting from the progressive loss of neurons in the compact part of the substantia nigra, located in the midbrain. 1

The worldwide prevalence of PD is around 150 to 300 per 1 million inhabitants, and 2 to 3% in individuals over 65-years-old, rising to 4 to 5% in the population over the age of 85 years. 2 A study in the municipality of Bambuí, in the state of Minas Gerais, Brazil, found a prevalence of 3.3% in individuals over 65-years-old. 3

Furthermore, PD triggers motor and non-motor signs/symptoms, and the decline in motor control results from alterations affecting the sensory-motor-cognitive systems. 4 Motor control, which includes postural and gait control, depends on the interaction of the sensory (e.g., somatosensory, visual, and vestibular) systems to maintain semi-static and dynamic postural stability, which are deficient in PD. 5 6

Changes in postural stability in PD have been widely studied, and various aspects of motor control are affected, leading to alterations in the execution of this control. 5 7 8 9 Studies of the kinematics and kinetics of gait and stepping have identified the struggles of PD patients with initiating stepping, demonstrated by anticipatory postural adjustment, hesitation, impairments in the spatiotemporal parameters of gait, and recurrent freezing of gait. 5 8 10 Falls are the most dangerous consequences of body imbalance. 11

Gray and Hildebrand 12 found that 40% of falls in a cohort of 118 PD patients resulted in physical, functional, and psychological impairment. The physical and psychological consequences of falls can lead to hospitalization, wheelchair dependency, and an incapacitating fear of falling, which can initiate a decline in patients' quality of life. It is estimated that people with PD fall frequently, with approximately 60% annually and around 39% recurrently. 13 14 The risk of falls is associated with different components in PD, such as increasing age, hypotension, and depression. 15 16 17 These alterations are essential characteristics of morbidity and mortality in this pathology.

The neural systems involved in postural instability in PD include impaired processing of neural signals in the substantia nigra and basal ganglia. 18 Other brainstem nuclei can contribute to this condition, including the vestibular ones and the pontine peduncle nucleus (PPN). 19 20

Among nonpharmacological treatments, noninvasive neuromodulation is a known tool that can stimulate and modulate neural centers involved in motor control alterations in this condition. For example, stimulation in the primary motor cortex, left dorsolateral frontal cortex, and the supplementary motor area seems to help treat gait in people with PD. 21 22 23

The vestibular apparatus is a sensory system that complements the corticospinal system, 24 and current scientific studies have proven its involvement in PD. 25 26 Noninvasive neuromodulation can stimulate the vestibular system through the use of galvanic vestibular stimulation (GVS), 27 which has been shown to modulate the production of neurotransmitters (dopamine, noradrenaline, and Gaba) in the circuits of the basal ganglia in animal studies. 18 28 29 Studies have identified that vestibular projections can stimulate the basal ganglia and the PPN, strengthening the neural networks involved in motor control. This may represent a gain in postural stability and a reduction in bradykinesia in PD patients.

Given this, it seems possible that vestibular signals integrate with other sensorimotor inputs in the striatum. This is the central area of vestibular stimuli in the basal ganglia, influencing motor control and clinical implications in treating related disorders. 25

The use of GVS can depolarize vestibular hair cells and first-order neurons, subsequently recruiting the neural pathway and stimulating frontal, temporal, parietal, and cerebellar regions. 27 30 The neural activity generated by GVS recruits the vestibulo-ocular and -spinal reflexes. Consequently, it increases postural stability, as shown in older adults at risk of falling and young people without alterations in body balance, with an improved effect on their postural performance. 31 32 33

Mahmud et al. 1 carried out a systematic review with meta-analysis of the use of GVS in postural balance in people with PD (n = 5) and concluded that it has a positive effect on postural balance. Still, the evidence must be more conclusive because the selected studies show low statistical power, heterogeneous stimulation parameters and methodologies, and a lack of randomization.

About the GVS method applied, the following varied: intensity, from 0 to 0.7 mA; electrodes placed on the mastoids, or on the mastoid and spinous process of the seventh cervical vertebra, or other placements such as mono or bipolar, and biocathode; and duration of stimulation, from 26 seconds to 3 hours. Furthermore, five articles included people with PD in the “on” phase and one article assessed the “on” and “off” phases. The disease's stages ranged from 1 to 4, following the Hoehn and Yahr scale (HY).

The primary limitations observed in the studies that have evaluated the effect of GVS in people with PD are: low number of participants; heterogeneity in clinical pictures (axial symptoms) and disease staging; low statistical power; heterogeneity of GVS parameters and methodologies; and lack of randomization and clinical trial according to the Consolidated Standards of Reporting Trials (CONSORT) guidelines for nonpharmacological interventions.

Additionally, the studies mentioned above did not analyze the effects of GVS associated with physical-functional exercise. 1 27 34 35 Although it has been described that head positioning can interfere with the postural response when a person is standing. Furthermore, the amplitude and direction of head rotation during GVS varies with its positioning, indicating that head movement and posture adopted during the GVS can interfere in the responses. 27

Therefore, the association of physical-functional exercise with GVS should be investigated in patients with PD, since it can potentiate the effects of the latter on motor control. This study has practical and scientific implications and is relevant due to the gaps highlighted in the literature regarding the use of GVS in people with PD and to deepen the knowledge of how this treatment affects motor control in this population. Additionally, the authors propose an innovative non-drug treatment for PD focused on noninvasive neuromodulation techniques that can influence the motor control centers involved in the pathophysiology of PD and minimize fall episodes.

In this context, our principal aim was to analyze the effects of GVS associated with physical-functional exercise on the motor control of PD patients. Our specific objectives were to analyze the effects of this treatment on body balance, gait, postural transfer, manual dexterity, physical capacity, confidence in body balance, risk of falling, and quality of life in patients with PD. Furthermore, we aim to analyze its effects on the safety and satisfaction of patients after treatment, as well as its permanent effects over 1 month.

Our hypotheses are that people with PD undergoing treatment with GVS associated with physical-functional exercise will show better motor control performance than those without GVS (placebo). Furthermore, they will perform better in body balance, gait, postural transfer, manual dexterity, and physical capacity tasks. We also expect an improvement in their quality of life, when compared with the placebo group. We believe GVS associated with physical-functional exercise is safe and satisfactory for PD patients, and that its effects will last approximately 1 month after treatment, with patients maintaining physical-functional exercise.

## METHODS

This study was approved by the Ethical Comitte of the Instituto de Assistência Médica ao Servidor Público Estadual de São Paulo – Hospital do Servidor Público Estadual (IAMSPE-HSPE), under the CAAE number 81822124.0.0000.5463. It was also registered on the Brazilian Clinical Trials Registry (ReBEC) under the number RBR-996f8qn, on February 10, 2025, as well as UTN U1111-1315-4982, on November 6, 2024.

### Study design

The present is a randomized, placebo-controlled clinical trial, which followed the CONSORT guidelines for nonpharmacological interventions.

### Study location

The clinical trials will take place in the Movement Disorder Department at HSPE.

### Inclusion and exclusion criteria

Patients between 50 and 75-years-age, female or male, with a diagnosis of idiopathic PD in stages 2 to 3 of the modified HY, regularly followed up at the Movement Disorder Outpatient Clinic and able to walk independently, will be included presenting in the “on period” and stable on their antiparkinsonian medication for 3 months.

Diagnosis will be based on the Brain Bank for Neurological Diseases criteria at the National Hospital for Neurology and Neurosurgery in London, which requires the presence of bradykinesia and at least one of the three cardinal signs of PD. 36

The exclusion criteria are patients unable to remain independently in the orthostatic position; severe visual impairment or not compensated for with the use of corrective lenses; orthopedic disorders resulting in movement limitations; use of medications affecting the vestibular system (e.g., cinnarizine, flunarizine, betahistine, dimenhydrinate, meclizine and ginkgo biloba, among others); having deep brain stimulation (DBS); a history of seizures, pacemakers, or metallic implants in the head and neck region; and those undergoing neurofunctional physiotherapy who did not agree to suspend this treatment during the study period.

Patients will be assessed in the “on” period, around 40 minutes to 2 hours after levodopa administration. The “on” state standardized for the test is the state in which the patient, according to their point of view, shows the best motor performance and is related to the effect of levodopa.

All patients will be assessed following the Head Impulse, Nystagmus, and Test of Skew (HINTS) evaluation to analyze vestibular function in them.

### Randomization

People with PD will be randomized according to the randomization website of the University of São Paulo (USP) separating the patients into two groups (Group A: Control, and B: Experimental). The researcher carrying out the interventions and the one in the assessments will stay blind about the randomization (double-blind study).

### Procedures

**Figure 1:**
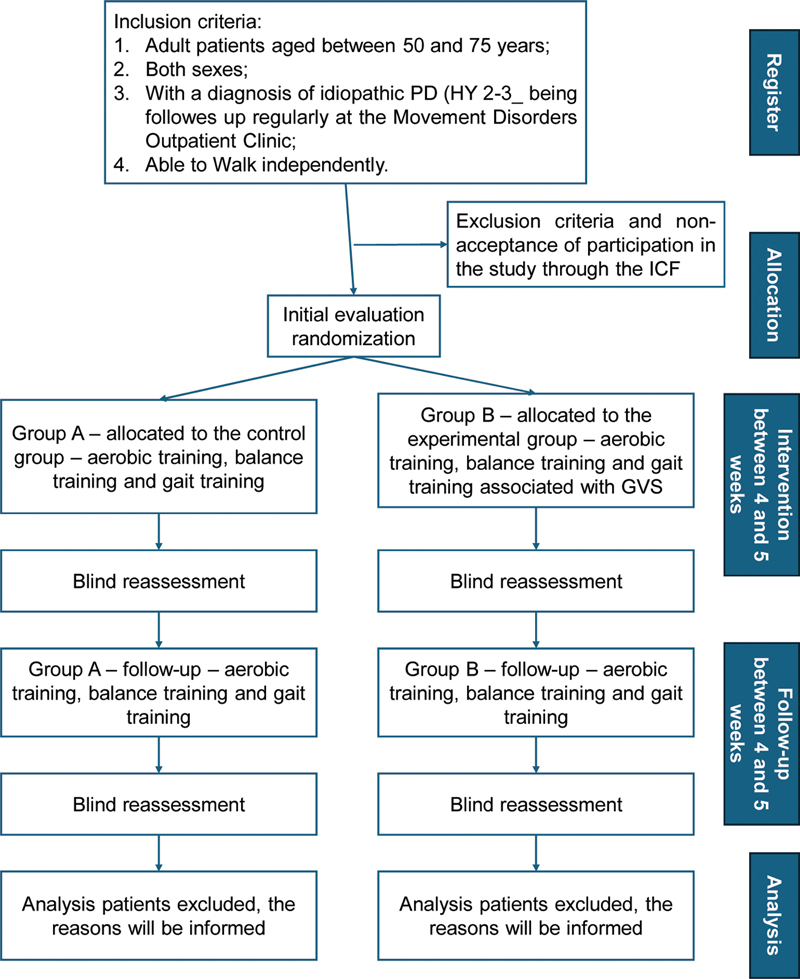
Abbreviations: PD, Parkinson's disease; HY, Hoehn and Yahr scale; ICF, informed consent form; GVS, galvanic vestibular stimulation. Flow diagram of the study project.

Figure 1 describes the phases of the study. Participants from both groups will be randomly allocated to the groups. The study will be conducted over 4 to 5 weeks, followed by follow-up.

### Outcomes measures

The primary outcome ( Table 1 ) for this research is body balance, measured through the mini-balance evaluation systems test (Mini-BESTest), as well as the activities-specific balance confidence scale (ABC Scale).

**Table 1:** Assessment tools for people with Parkinson's disease recommended by the European Physiotherapy Guideline for Parkinson's Disease 39

Outcome	Instrument
Body balance	Mini-BESTest
Locomotion	Spatio-temporal gait parameters, NFoG-Q
Postural transfer	M-PAS
Manual dexterity	Nine Holes and Pins Test
Physical capacity	6MWD with Borg 6-20, FTSTS
Confidence in body balance	ABC Scale
Fall risk	Mini-BESTest
Disease severity	UPDRS
Quality of life	PDQ-39
Safety and satisfaction	Questionnaires on adverse effects and satisfaction

Abbreviations: 6MWD, six-minute walk; ABC, activities-specific balance confidence scale; Borg 6-20, Borg scale 6-20; FTSTS, five times sit to stand; M-PAS, modified Parkinson activity scale; Mini-BESTest, mini-balance evaluation systems test; NFoG-Q, new freezing of gait questionnaire; PDQ-39, Parkinson disease questionary-39; UPDRS, unified Parkinson's disease rating scale (part III – motor).

The secondary outcomes were spatio-temporal gait parameters measured using the BTS G-walk (Kinetec) inertial sensor system. Freezing of gait, measured with the new freezing of gait questionnaire. Postural transfer, measured with the timed up and go test and the modified Parkinson activity scale (M-PAS). Manual dexterity, with the nine holes and pins test. Physical capacity, measured with the six-minute walk (6MW) and five times sit to stand tests. Furthermore, risk of falling was measured through the Mini-BESTest, with a cut-off point of 16. 37 Disease severity was measures through part III (motor) of the Unified Parkinson's disease rating scale (UPDRS). Quality of life was analyzed with the Parkinson disease questionary-39 (PDQ-39). 38 Finally, patients' safety and satisfaction will be determined through a new questionnaire, in which adverse events and participants' satisfaction after treatment will be recorded.

### Preintervention, postintervention, and follow-up evaluation protocol

The pre- and post-intervention, as well as follow-up evaluation protocol has been prepared and is available with all the instruments used. The assessment procedures follow the European Physiotherapy Guideline for Parkinson's Disease. 39 The assessment will last 1 to 2 hours.

### Intervention

The stimulation period will include ten sessions of GVS associated with physical-functional exercise according to the American Guideline for the Management of Parkinson's Disease. 40 Patients were monitored by our team during aerobic, postural balance, and gait exercises. These three items are of high-quality evidence and muscular strength of recommendation, according to the American Physical Therapy Guideline for the Management of Parkinson's Disease. 40 The series of exercises will be standardized.

### Physical-functional exercises

Physical-functional exercises will be protocolized. Three blocks will be performed, warm-up, after it will be performed visuo-vestibular exercises and finally, gait training. More specific, patients will be asked to stay in orthostatism posture inside a parallel bar to provide security. Then, they will be asked to perform visual fixation (10 times, 10 s each), smooth pursuit (10 series, 10 trials each), adaptation vestibular exercises with visual fixation during horizontal and vertical head movement (10 series of 10 trials each, in sequence).

Finally, there will be gait training (10 minutes) with anterior, lateral, and posterior gait, as well as walking and moving the head to the sides and up and down with their gaze fixed on a target ahead, and passing through and step.

Each block of exercises should last 10 minutes, leading to a total of 30 minutes of physical-functional exercises.

#### *Current and dosimetry of GVS*

Dosimetry will be uniform between patients, using noise-type galvanic vestibular current (stochastic phenomenon), with an intensity of 0.7 mA; frequency 1 of 2 Hz and frequency 2 of 20 Hz. Intensities below 1.0 mA can recruit vestibular hair cells and thus progress the pathway as a whole. Furthermore, these frequencies are related to activity in the muscles of the head, trunk, and lower limbs, as well as to postural oscillation, vestibular system activity. Finally, a 20 second upward ramp and 20 second downward ramp, with a total duration of 30 minutes. 20 27 The effective blinding will be ensured for participants with a brief ramp-up current at the start, without sustained stimulation.

#### *Positioning the electrodes for GVS*

The current must be delivered through silicone electrodes (5 × 7 cm) positioned on the mastoids people with PD, keeping the cathode on the right and the anode on the left. However, the current type is not polarized in a single direction. The electrodes are soaked in 0.9% saline solution and fixed to the head using an elastic band.

#### *Galvanic vestibular stimulation device*

The device used is the Microestim FOCO Research (NKL) stimulator, purchased specifically for the research, with silicone electrodes, coupling sponges, and cables.

#### *Sample size calculation*

The study groups should contain 15 patients each. The sample calculation considered how long each patient followed the protocol (∼ 2 months). We found no previous studies with the same outcomes and intervention protocol.

### Statistical analysis

Initially, descriptive statistics should be used to analyze the data. To this end, summary measures must be calculated, followed by tables appropriate to the nature and measurement level of the variables involved.

The comparison of groups and moments in time throughout the intervention will be done after the verification with normality tests. If data were normally distributed, the repeat measures analysis of variance (ANOVA) will be used to compare groups (placebo and GVS) over time. If not normally distributed, we will use Freidmans's test for non-parametric data. Furthermore, the ANOVA test will be applied to assess the behavior of the quantitative variables before and after treatment and follow-up between groups, and repeated measures ANOVA will be implemented for effect within groups over consecutive treatment sessions. The significance level adopted for the statistical tests will be 5% (a = 0.05). Analyses and graphs will be done using the Statistical Package Social Sciences (SPSS Inc.) for Windows, version 10.0.

### Ethical considerations

Before starting any research stage, participants will be fully informed verbally and in writing about all the stages of the research and its importance. They are free to accept or not to participate in the study. All participants must sign an informed consent form (ICF).

Participants will not be charged any fees or bonuses for participating and must be aware of their scientific contribution. The researchers will not disclose participants' data or the images captured on video. The data will be used exclusively for scientific purposes.

This research aims to promote new knowledge for professionals in this field. There will be direct benefits for patients, as they will be assessed and treated by an interdisciplinary team to reduce/remove body imbalance, gait alterations, and the risk of falls.

During the assessment and treatment, patients may experience dizziness, body imbalance, and difficulty walking. However, a researcher will always remain close to each patient to avoid falls and adapt the exercises according to their needs and threshold for dizziness and sensory-cognitive-motor difficulties. There will always be two examiners for the gait assessment.

The risks of GVS are related to its adverse effects: itching and discomfort at the stimulation site, as well as local burning. These can be minimized by thoroughly soaking the sponges to attach the silicone electrodes to the skin behind the ears in the mastoid area.
